# Stability in the face of global decline: a 20-year study of arthropods in an oceanic archipelago

**DOI:** 10.1038/s41598-026-57146-5

**Published:** 2026-06-17

**Authors:** Gabor Pozsgai, Pedro Cardoso, Simone Fattorini, François Rigal, Ana M. C. Santos, Robert J. Whittaker, Isabel R. Amorim, Mário Boieiro, Luís Borda-de-Água, Ricardo Costa, Luís Carlos Crespo, Maria Teresa Ferreira, Abrão Leite, Sébastien Lhoumeau, Jagoba Malumbres-Olarte, Thomas J. Matthews, Catarina Melo, Guilherme Oyarzabal, Fernando Pereira, José A. Quartau, Carla Rego, Sérvio Ribeiro, Alejandra Ros-Prieto, Artur R. M. Serrano, Kostas A. Triantis, Rosalina Gabriel, Paulo A. V. Borges

**Affiliations:** 1 Centre for Ecology, Evolution and Environmental Changes CHANGE Global Change and Sustainability Institute, University of the Azores, Rua Capitão João d´Ávila, Pico da Urze, 9700-042 Angra do Heroísmo, Portugal; 2 CE3C Centre for Ecology, Evolution and Environmental Changes, Change Global Change and Sustainability Institute, Faculty of Sciences, University of Lisbon, Lisbon, Portugal; 3 LIBRe Laboratory for Integrative Biodiversity Research, Finnish Museum of Natural History, University of Helsinki, (Pohjoinen Rautatiekatu 13), P.O.Box 17, 00014 Helsinki, Finland; 4 Department of Life, Health and Environmental Sciences, University of L’Aquila, Via Vetoio, 67100 L’Aquila, Italy; 5 Institut Des Sciences Analytiques et de Physico Chimie pour L’environnement et les Materiaux, UMR5254, Comité National de la Recherche Scientifique - University de Pau et des Pays de l’Adour - E2S UPPA, Pau, France; 6 Terrestrial Ecology Group (TEG-UAM), Departamento de Ecología, Universidad Autónoma de Madrid, 28049 Madrid, Spain; 7 Centro de Investigación en Biodiversidad y Cambio Global (CIBC-UAM), Universidad Autónoma de Madrid, 28049 Madrid, Spain; 8 School of Geography and the Environment, University of Oxford, South Parks Road, Oxford, OX1 3QY UK; 9 Center for Macroecology, Evolution and Climate, GLOBE Institute, University of Copenhagen, Universitetsparken 15, 2100 Copenhagen, Denmark; 10 IUCN SSC Atlantic Islands Invertebrates Specialist Group, University of the Azores, 9700-042 Angra do Heroísmo, Azores Portugal; 11 CIBIO Centro de Investigação em Biodiversidade e Recursos Genéticos InBIO Laboratório Associado Campus de Vairão, Universidade do Porto, 4485-661 Vairão, Portugal; 12 CIBIO Centro de Investigação em Biodiversidade e Recursos GenéticosInBIO Laboratório Associado, Instituto Superior de AgronomiaUniversidade de Lisboa, 1349-017 Lisboa, Portugal; 13 BIOPOLIS Program in Genomics, Biodiversity and Land Planning, CIBIO Campus de Vairão, Universidade do Porto, 4485-661 Vairão, Portugal; 14 Regional Secretariat of Environment and Climate Action, Universidade dos Açores, Rua do Galo n118, 9700-040 Angra do Heroísmo, Azores Portugal; 15 Escola Superior de Hotelaria e Turismo do Estoril, Rua Fernando Pessoa, nº99 R/C DTO, 2765-483 Estoril, Portugal; 16 GEES School of Geography, Earth and Environmental Sciences and Birmingham Institute of Forest Research, University of Birmingham, Birmingham, B15 2TT UK; 17 Laboratório de Ecologia do Adoecimento e Florestas, NUPEB, Instituto de Ciências Exatas e Biológicas Campus Morro do Cruzeiro, Universidade Federal de Ouro Preto, Ouro Preto, MG 35400-000 Brazil; 18 Department of Ecology and Taxonomy, Faculty of Biology, National and Kapodistrian University of Athens, GR-15784 Athens, Greece; 19 IUCN SSC Species Monitoring Specialist Group, University of the Azores, 9700-042 Angra do Heroísmo, Azores Portugal

**Keywords:** Ecology, Ecology, Zoology

## Abstract

Insect declines are of global concern, yet no long-term ecological studies (LTER) have confirmed this trend on islands. This study utilises the first available LTER data on island arthropods, targeting epigeal and canopy species from the Azores Archipelago (Portugal), and covering over 20 years in three distinct sampling events from 30 standard sites. We investigate changes in abundance, biomass, and species richness within native forest arthropod communities, focusing on the proportions of endemic and introduced species, and temporal patterns among single-island endemics and forest-dependent endemics. Results reveal significant temporal variability, but overall abundance, biomass, and species richness remain stable across endemic and native non-endemic taxa. Among the species studied, 28% declined, 17% increased, and 55% showed no significant differences. Exotic invasions and related extinctions appear minimal. Forest-dependent endemic species declined below anticipated levels, suggesting that the extinction debt for these species may be less severe than initially expected. Nonetheless, some forest specialists have declined significantly, and seven species, not seen over 20 years, are considered to be extinct. The three-decade-long conservation of Azorean native forests may have contributed to the stability of some populations, thus these findings underscore the need for continued and enhanced conservation efforts of insular forest-associated diversity.

**Supplementary Information:**

The online version contains supplementary material available at https://doi.org/10.1038/s41598-026-57146-5.

## Introduction

Collectively, oceanic islands harbour disproportionate amounts of biodiversity and in particular, high proportions of endemics compared to continents^1^. However, in addition, oceanic islands also possess some of the world’s most threatened biotas^1^. Their vulnerability stems from high levels of anthropogenic exploitation, habitat degradation, and the deleterious influence of introduced species relative to their land area^2,3^, which is further exacerbated by the effects of ongoing climate change^4^ and the elevated vulnerability of species evolved in isolation^5^. Consequently, the rate of historical and contemporary anthropogenic extinction on islands surpasses that of the continents^6^.

Arthropods are fundamental elements of most ecosystems and several recent studies have reported declines^7,8^, particularly in relation to insects. Arthropod declines may lead to an accelerating deterioration of ecological functions provided by these organisms^9^, diminishing ecosystem services (e.g. pollination, regulation of pests, decomposition) and, ultimately, leading to ecosystem collapse^10^. Despite several studies raising the alarm in terms of insect declines^7,11,12^, insect populations do not show a consistently declining pattern, particularly as trends have been found to differ between taxa and functional groups and habitat type^12^. As is true for other taxonomic groups, some species may increase in prevalence^8^ and the proportion of winners often counterbalances that of the losers (Following Dornelas et al.^13^, ‘winners’ and ‘losers’ denote populations that, respectively, increase or decline in abundance and occupancy through time.).

While island biotas are known to be particularly threatened, few studies have tested for insular arthropod declines through time. Thus, the extent to which islands are implicated in global insect declines remains unknown. Island endemics in particular are expected to decline^1^, with many rare endemic species expected to be threatened with extinction. Yet, largely due to the scarcity of systematic, standardised sampling and long-term datasets for insular arthropods, few of these predictions have been rigorously tested, limiting our ability to assess their generality for remote island arthropods. Moreover, since the rate of non-native species introductions on islands may exceed the extinction of endemic species^14^, it is key to gain understanding into whether population trends of endemic, native but not endemic (henceforth native), and introduced (or exotic) species follow similar trajectories.

The humid forests of the Azorean Islands (North Atlantic, Macaronesia) form a distinctive habitat type that once covered ~99% of the archipelago. By the second half of the 20^th^ century its area had shrunk to its current range of only 5% of its original size^15^. Due to the legal protection of the most pristine areas, the current area of the forest remnants has not decreased for almost four decades and direct human disturbances have been reduced significantly. Yet, the persisting forest patches are highly fragmented and prone to indirect (mostly anthropogenic) influences, such as the spread of exotic, and potentially invasive, species^16^ and climate change^17–19^. Moreover, it remains unclear whether these forest patches are large enough to sustain viable populations of endemic forest-dependent arthropod species and halt the future extinction processes that have been predicted^2,16^. Hence, our focus herein is primarily on the endemic species that survive in less-than-ideal circumstances, some of which may not present viable populations, contributing to so-called extinction debt (extinctions delayed after habitat loss or environmental change)^20^. Notwithstanding the apparent urgency of comprehensive conservation strategies for these imperilled species^21,22^, the lack of in-depth insight into population trends (Prestonian shortfall^23,24^) of insular arthropods may hamper the development of effective protection measures.

**Fig. 1:**
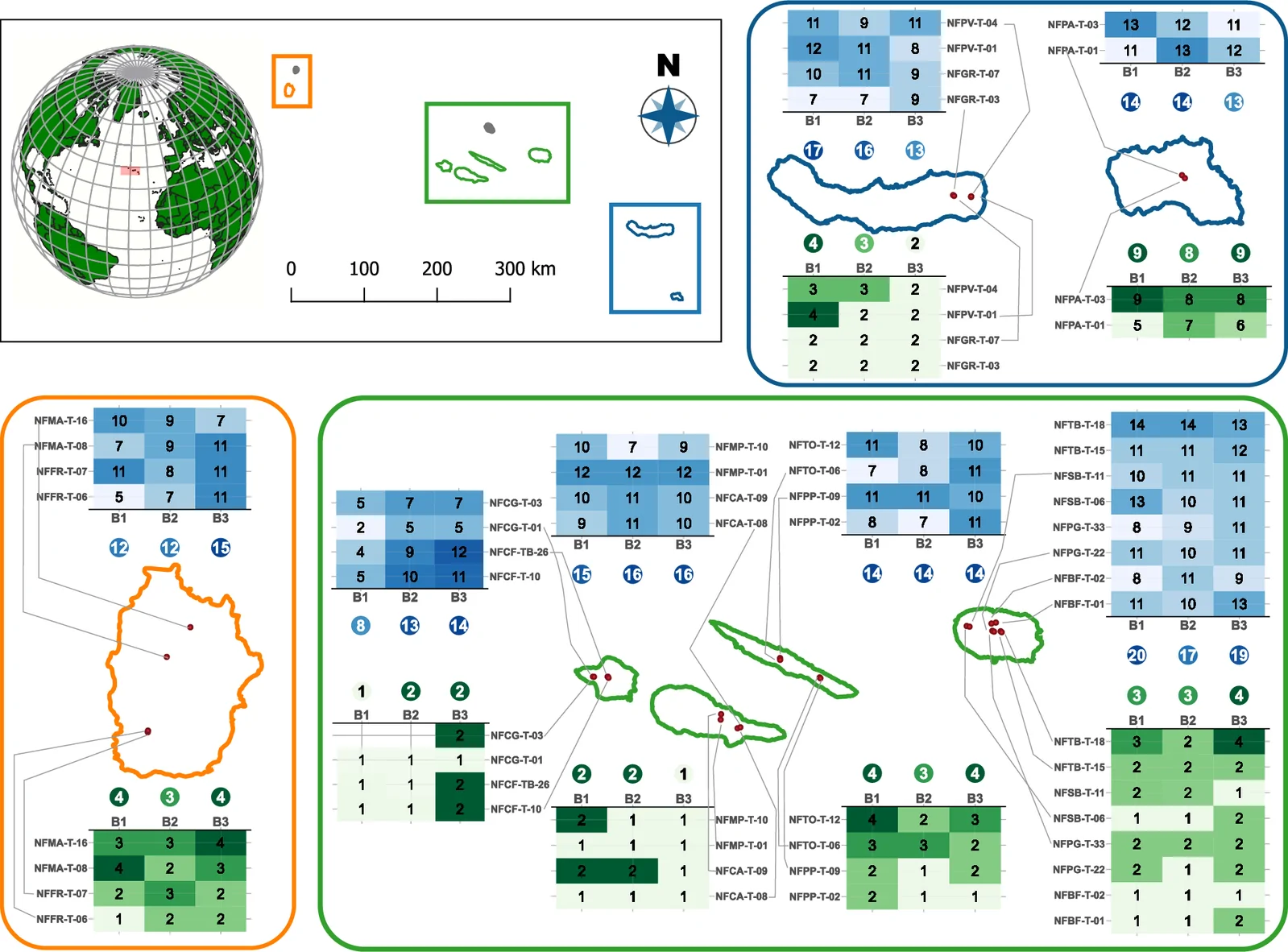
The location of the Azorean Archipelago and the seven sampled islands (top left) in the three island groups (colour-coded as orange, green, and blue for the Western, Central, and Eastern groups, respectively). Non-sampled islands coloured grey. Sampling sites in the three detailed subfigures are indicated with red dots, and arrows linking them to rows in the blue and green tables show the number of single island endemic species (SIE) or strict forest-dependent endemics (FDE) (respectively) in each sampling campaign. Numbers in circles represent the number of unique SIE/FDE species caught in each BALA sampling campaign (B1-B3) from the entire island. Abbreviations as: FLO – Flores, FAI – Faial, PIC – Pico, SJG – São Jorge, TER – Terceira, SMG – São Miguel, and SMR – Santa Maria.

In this study, we utilise a dataset of standardised samples collected in 1999-2000, 2010-2011, and 2020-2023. This unique data series, compiled as part of the “Biodiversity of Arthropods from the Laurisilva of the Azores” (BALA) project^25^, allows us to address a fundamental question: are arthropods declining in the native forests of remote oceanic islands? To answer this, we pay particular attention to species of outstanding conservation importance, such as single-island endemics (SIE) and strict forest-dependent Azorean endemics (FDE) considered to be either already extinct^2^ or to be facing extinction debts^20,26^ (Fig. 1), while also scrutinising how exotics change over time in abundance and species richness. Moreover, because overall temporal patterns of arthropod abundances may be differentially influenced by island-wide trends, we also ask how each individual island contributes to these overall changes, employing a simulation approach to investigate. Our dataset holds a collection of abundance data of ground-dwelling (epigeal henceforth, collected with pitfall traps) and canopy-dwelling (arboreal henceforth, collected by canopy beating) arthropods from 30 repeatedly sampled sites across seven Azorean islands, collected in three distinct archipelago-wide sampling campaigns (B1, B2, and B3, henceforth) from 1999 to 2023.

We found no significant overall decline in arthropod abundance, biomass, or species richness in native Azorean forests from 1999 to 2023, although moderate losses were observed in ground-dwelling communities, especially among native and endemic species. Whilst few single-island and forest-dependent endemic species declined, others increased or remained stable, contradicting earlier predictions of a widespread extinction.

## Results

### Changes in overall abundance

The data encompass about 17% of the known Azorean arthropod species described to date, including ~33% of known archipelagic endemic taxa from 15 native forest fragments^27–29^. This corresponds to 30,370 observations of 405 arthropod species (of which 91 or ~22% are archipelagic endemics and 155 or ~38% introduced, see also Pozsgai et al.^25^).

To assess the yearly changes in overall arthropod abundances between the sampling events, we first calculated the average differences for the years between consecutive sampling events. Using Cohen’s d and bootstrapped confidence intervals, we determined whether standardized effect sizes of transect-wise changes significantly diverged from zero.

**Fig. 2:**
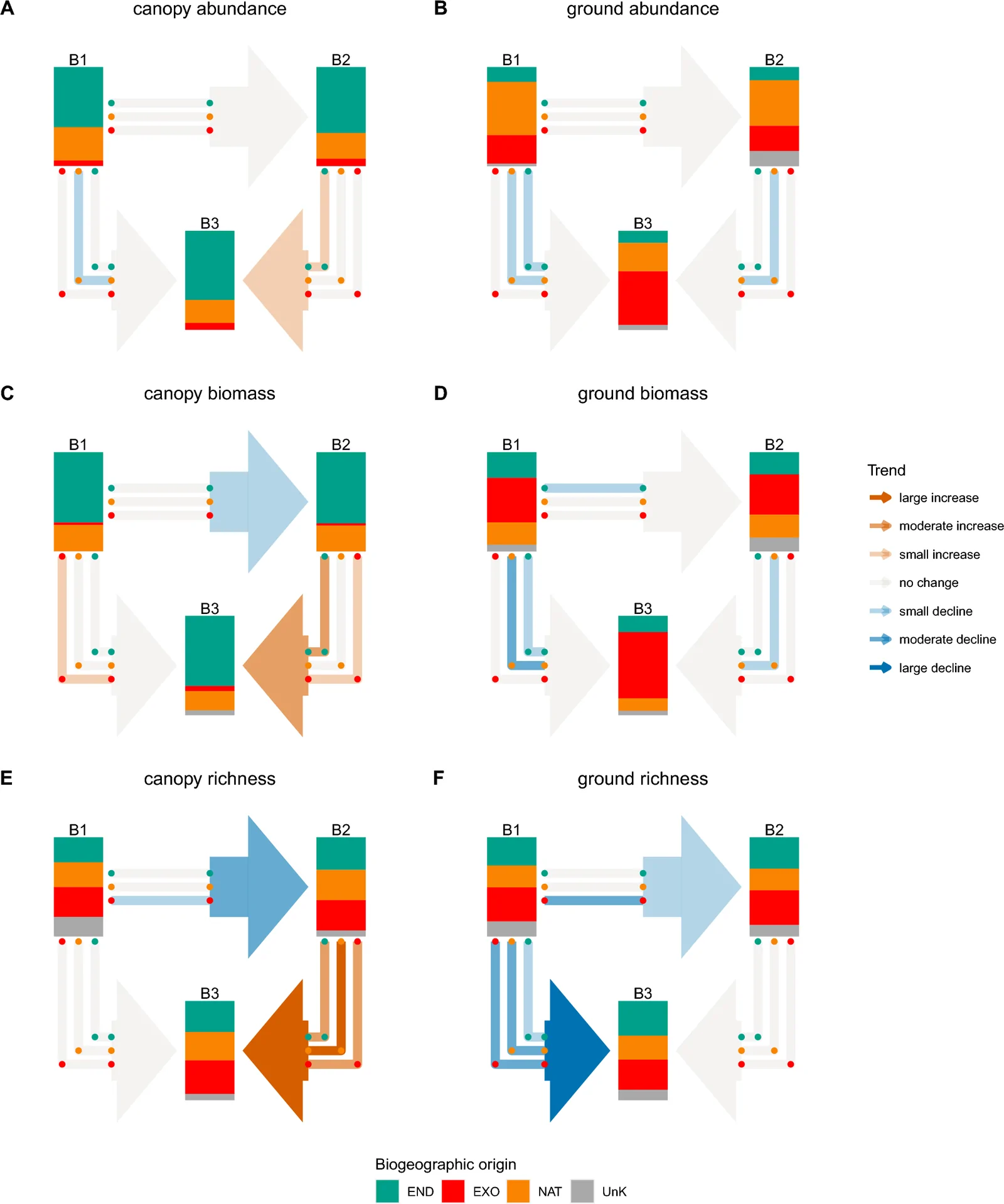
Summary of changes in abundance (**A**-**B**), biomass (**C**-**D**), and richness (**E**-**F**) between BALA sampling campaigns (B1-B3) in arboreal (**A**, **C**, **E**) and epigeal (**B**, **D**, **F**) communities. The proportion (from top to bottom) of endemic (END), non-endemic native (NAT), introduced (EXO) species, and those with unknown biogeographic origin (UnK) in the communities are shown in the barplots. The colours in the barplots, representing biogeographic origins (refer to legend), also correspond to the dots along the thin lines that indicate changes for each group (excluding unknown origins). The arrows indicate the direction of comparison and their colour indicates the overall changes. Changes are standardised to time unit (year) and compared to a ‘zero change’ null model. Change was considered statistically not significant when confidence intervals crossed zero. The magnitude of change is indicated with colour depth, increasing from small (representing a Cohen’s d value between 0.2 and 0.5), through medium (Cohen’s d value between 0.5-0.7) to a large change (Cohen’s d > 0.7).

Our findings indicated no significant overall change in arthropod abundance (i.e. when endemics, natives, and exotics, were all included), between the initial (B1) and final (B3) sampling campaigns conducted from 1999 to 2023, in either ground- or canopy-dwelling communities (Fig 2 A-B). However, notable differences were observed between B1 and B2, and B2 and B3, as well as among species with different biogeographic origins. A small decrease from B1 to B3 was seen for non-endemic native species, both in ground and arboreal communities, with Cohen’s d values of -0.48 (CI: [-0.84, -0.36]) and -0.32 (CI: [-0.55, -0.03]), respectively (Supplementary Table 1). Ground-dwelling endemic species also exhibited slight reductions in abundance (d = -0.47, CI: [-0.83, -0.12]). However, the abundance of arboreal endemic species increased from B2 to B3, with a Cohen’s d of 0.39 (CI: [0.02, 0.94]) (Fig 2 A-B). The proportions of endemics and exotics relative to the overall number of individuals remained unchanged between B1 and B2 (Supplementary Table 1, Supplementary Figure 1 A-B).

### Changes in biomass

Since body mass is a disproportionately important trait driving ecosystem functions and it is often regarded as a superior currency in terrestrial invertebrate community analysis^30^, we also analysed changes in arthropod biomass. Despite estimating biomass using taxon-specific indices and individual counts (see Methods), the correlation between overall biomass and abundance was not very strong (Pearson’s ρ = 0.63, p < 0.001). Overall canopy biomass exhibited small-magnitude losses between B1 and B2 which were offset by gains from B2 to B3, resulting in no net changes in overall biomass across the over 20 years but showing a small increase in the biomass of arboreal exotic species (d = 0.41, CI: [0.21, 0.64]) (Fig 2 C-D). In ground-dwelling communities, biomass differences closely mirrored abundance patterns and showed no significant changes between either sampling campaign. However, when groups of different biogeographic origin were examined separately, the biomass of both endemic and native ground fauna showed a decline between B1 and B3 (d = -0.32, CI: [-0.66, -0.09] and d = -0.67, CI: [-1.27, -0.51], respectively) (Fig 2 C-D, Supplementary Table 1). The increase of exotics in the canopy-dwelling community and the decline of endemic and native species in the ground-dwelling community significantly elevated the biomass-proportion of exotics in both cases from B1 to B3 (Supplementary Figure 1, Supplementary Table 1).

### Changes in species richness

Small and moderate declines in species richness were noted for both epigeal and arboreal arthropods between B1 and B2, with Cohen’s d values of -0.46 (CI: [-1.15, -0.07]) and -0.69 (CI: [-1.23, -0.31]), respectively (Fig 2 E-F, Supplementary Table 1). This was followed by a substantial increase in canopy-dwelling species from B2 to B3 (d = 1.13, CI: [0.75, 1.79]), resulting in no net change in arboreal species richness between B1 and B3. Conversely, ground-dwelling species showed no significant increases between B2 and B3, leading to declines in species richness from B1 to B3 (d = -0.80, CI: [-1.30, -0.44]).

When species groups of different biogeographic origins were analysed separately, ground-dwelling natives and exotics experienced greater losses from B1 to B3 (d = -0.56, CI: [-0.95, -0.21] and d = -0.68, CI: [-1.24, -0.31], respectively) compared to Azorean endemics (d = -0.47, CI: [-1.20, -0.08]). Arboreal native species showed the largest increase between B2 and B3. Species-richness proportions of endemics and natives relative to total richness remained unchanged throughout the study (Supplementary Table 1, Supplementary Figure 1).

### Changes in individual species abundances

Our second approach focused on the abundance changes of individual species, particularly those of high conservation concern, namely single-island endemics and strict forest-dependent endemics^20,26^. Of the 405 species present in our dataset, 84 (~21%) were sufficiently abundant to comply with our criteria for formal analysis (see Methods). For these, we used the same methodology as above to investigate how the abundances of individual species changed from B1 to B2 and B3, and from B2 to B3. Of these, 22 species declined significantly from B1 to B3, including 3 with small declines (Cohen’s d between 0.2 and 0.5), 13 with moderate declines (Cohen’s d between 0.5 and 0.7), and 6 with large declines (Cohen’s d > 0.7). The mean Cohen’s d for declines was -0.74 (range: -1.23 to -0.39). In contrast, 5, 7, and 1 species showed small, moderate, and large increases, respectively, with magnitude comparable to that of the declines (mean Cohen’s d = 0.67, range: [0.36, 2.20]).

**Fig. 3:**
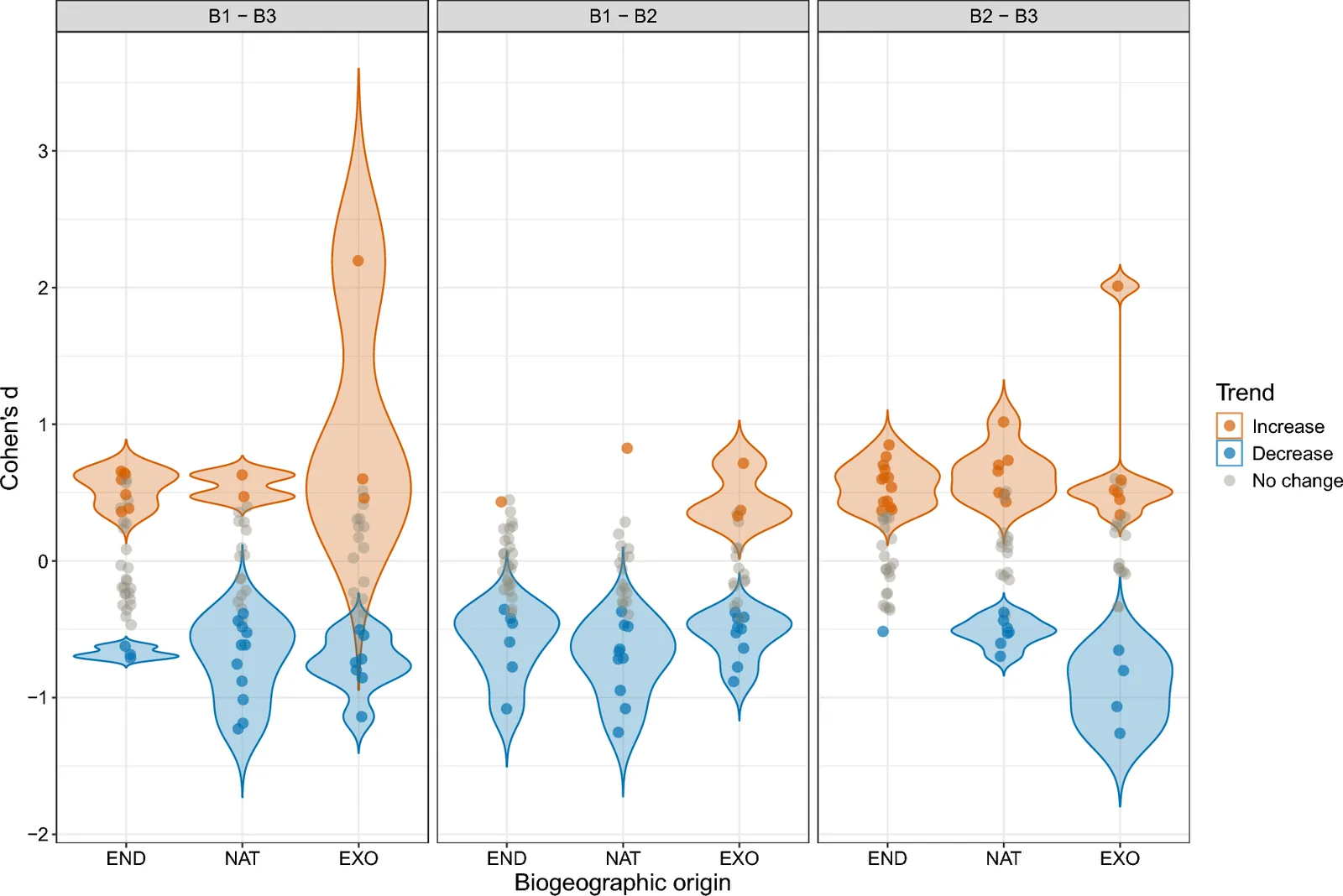
Mean change across transects in the abundances of Azorean native forest arthropod species between sampling campaigns. Changes are standardised to time unit (year) and compared to a ‘zero change’ null model. Species experiencing no significant changes (i.e. confidence intervals cross zero) are marked in grey. Violin plots indicate the frequency distribution of data points (each dot represents a species). END stands for endemic species, NAT for native but non-endemic, and EXO for introduced species. Only species whose abundance reached the requirements for the statistical analysis are included in the figure.

For endemic species, the mean effect size for overall decline (between B1 and B3) was -0.67 (range: [-0.71, -0.62]), while the increase was recorded at 0.54 (range: [0.36, 0.66]). Introduced species showed a decline with a mean effect size of -0.76 (range: [-1.14, -0.50]) and an increase of 1.09 (range: [0.46, 2.20]) (Fig 3.).

For species whose abundance was too low to allow individual temporal patterns to be analysed, we merely compared the sum of collected individuals from each sampling campaign and no statistical tests were conducted to compare the sampling campaigns. Here, 186 (~58%) showed declines, 39 (~12%) showed no change (i.e. no more than 20% difference between the first and last sampling events), and 96 (~30%) increased. Endemics and natives accounted for ~36% of declines, while exotics made up ~38%. No significant differences were found when the proportion of increasing and decreasing species based on biogeographic origin was compared (two-sided Fisher test p = 0.204). There were also no differences among the different biogeographic origins in the number of species increasing, being stable, or decreasing in the number of occupied sites (see Methods and Supplementary Table 8).

### Changes in individual FDE and SIE species abundances

**Fig. 4:**
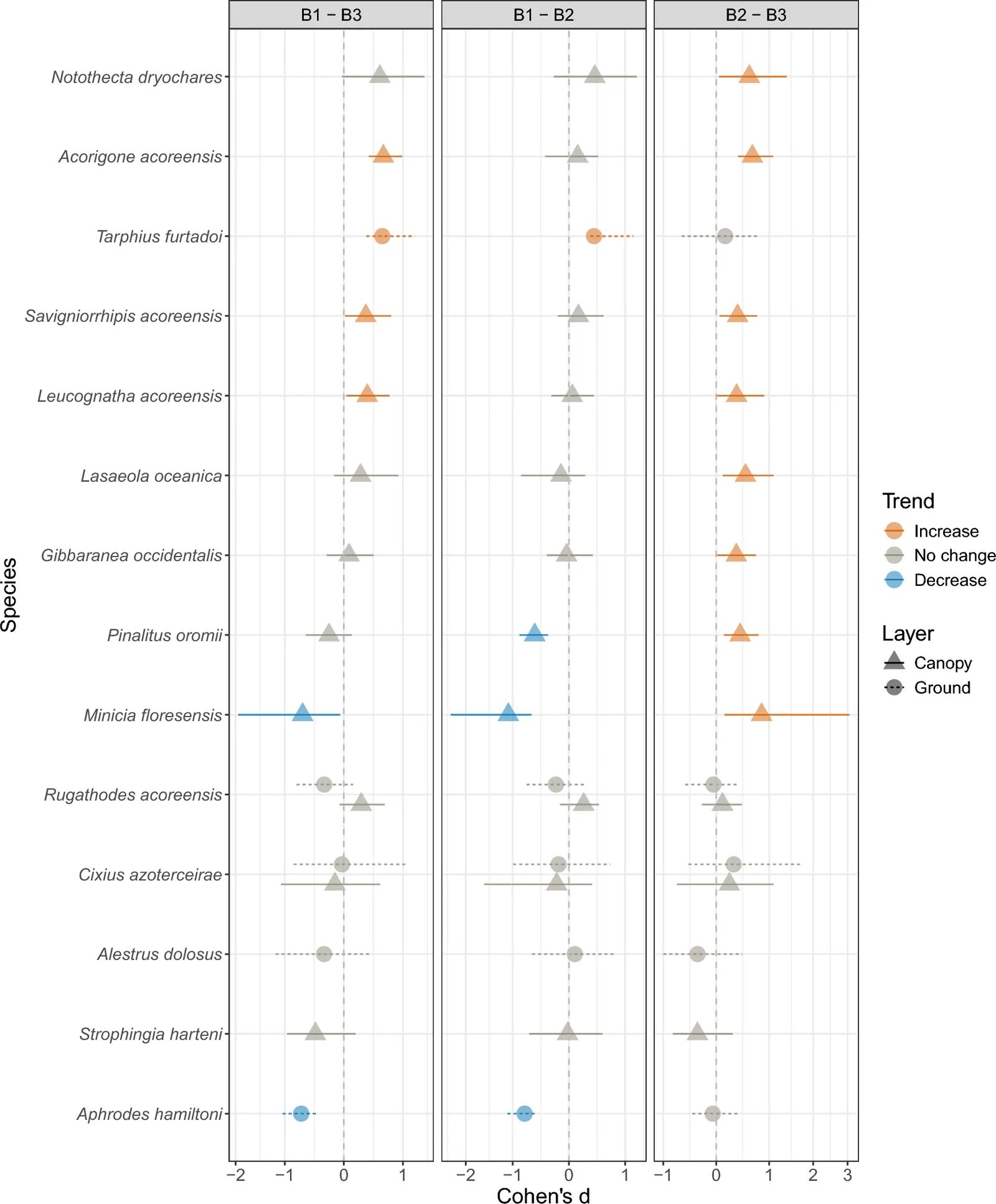
Abundance changes for strict forest-dependent endemics (FDE) from BALA 1 to BALA 3 (first panel), BALA 1 to BALA 2, and BALA 2 to BALA 3 sampling campaigns. Symbols indicate the mean log-transformed Cohen d-value and horizontal lines the corresponding confidence intervals. Statistically not significant (i.e. confidence intervals cross zero) changes indicated with grey colour. Labels on the x-axis are back-transformed and show mean annual changes in the number of individuals.

In our samples, 30 SIEs and 34 FDEs were found (Supplementary Table 2), and of these 14 FDEs meet our criteria for the calculation of reliable effect sizes and estimation of temporal differences between sampling campaigns (see Methods). The only SIE with sufficient data for testing, *Cixius azoterceirae* Remane & Asche, 1979, also appears as an FDE. The abundance patterns for FDE species mirrored overall abundance patterns: marked declines between B1 and B2 were followed by increases between B2 and B3. Indeed, while three FDE species declined moderately from B1 to B2 and one increased, seven species showed small to large increases between B2 and B3 without declines. Overall, two species of FDE, the leafhopper *Aphrodes hamiltoni* Quartau & Borges, 2003 and the money spider *Minicia floresensis* Wunderlich, 1992, showed moderate declines between B1 and B3, whereas four experienced small to large increases in abundance (Fig. 4, Supplementary Table 2).

Of those FDE and SIE species that did not meet our criteria for statistical analysis (see Methods), 25 became less numerous (by more than 20% of the original abundance) from B1 to B3, 4 showed no change, and 12 increased (Supplementary Tables 4-7). The proportions of these outcomes did not significantly differ from non-SIE or non-FDE species (two-sided Fisher test p > 0.05). However, the proportion of species decreasing in the number of occupied sites was significantly lower for FDE/SIE species compared to non-FDE/SIE species and exotics (one-sided Fisher tests, p = 0.015 and p = 0.048, respectively, Supplementary Table 8).

To explore how individual islands contributed to overall temporal differences in abundance, biomass, and richness, we used a simulation approach, removing one island at a time from the dataset or omitting combinations of two or three islands, and recalculating differences between B1 and B3 (see Methods).

These analyses showed that Terceira positively contributed to changes in overall abundance and richness of arboreal species, particularly endemics, while negatively contributing to the experienced differences in arboreal exotic species. Other islands had varying effects; for example, São Jorge positively contributed to endemic species in epigeal communities but added to declines in canopy arthropods and Santa Maria samples generally made a positive contribution to exotic abundances and richness (Supplementary Figures 2-10).

## Discussion

Understanding population trends of arthropods in insular biotas is essential for effective protection and the preservation of the ecosystem services that they provide, as well as to fully answer the challenges of global insect declines. In this study, we found minimal evidence of large-scale depletion in the arthropod fauna of Azorean native forests since the start of sampling (in 1999/2000) and neither did we observe significant declines in either endemic or non-endemic but native arthropod populations. Instead, we found highly variable abundances, biomasses, and species richness between sampling events in both epigeal and arboreal communities, with a balance of gains and losses over time. This finding adds nuance to the prevailing narrative of widespread insect decline worldwide and is consistent with other large-scale analyses showing that insect populations can exhibit declines, stability, or fluctuations depending on the taxa, habitats and regions considered^31–33^. Since endemics dominate in abundance over natives and exotics in the arboreal communities in the Azores^15,34^, this unchanged abundance pattern was driven by the relative stability of canopy endemic species. Similarly stable populations of endemic flying insects have been reported from Terceira Island over 6 and 10 year study periods^16,35^. In our study, even the species predicted to decline the most—such as single island endemics and forest-dependent endemics—generally remained stable, with some canopy-specialised endemic species, like the spider *Acorigone acoreensis* (Wunderlich, 1992), even experiencing population increases between the first and last sampling campaigns. Moreover, site occupancy for both forest-dependent (FDE) and single island endemics (SIE) did not significantly change and even showed slight increases, indicating at least some degree of habitat stability.

However, a degree of pessimism is warranted, as none of the beetle species previously classified as extinct by Terzopoulou et al.^2^ were detected during the three BALA surveys. According to their calculations for beetles, the extinction rate is estimated at 4.96 species per century. This translates to an expected loss of approximately 0.992 species over the 20 years of the BALA surveys or 1.24 species over 25 years. Notably, during the BALA study, at least two species, the ground beetle *Calathus lundbladi* Colas, 1938 endemic to São Miguel and the ironclad beetle *Tarphius serranoi* Borges, 1991 endemic to Santa Maria, meet the IUCN criteria for extinction, suggesting that the extinction rates predicted by Terzopoulou et al.^2^ appear to be corroborated. Indeed, the current protected remnants of Azorean native forests face some disturbances, including the aggressive spread of the invasive ginger lily (*Hedychium gardnerianum* Sheppard ex Ker Gawl.)^36^, which alters the edaphic conditions and microclimate of the forest understorey and inhibits the regeneration of indigenous flora^37^. The invasion by ginger lily may still be linked to negative trends we observed in epigeous species richness and declines in specialist herbivorous species like *A. hamiltoni*. However, excluding São Miguel from our analysis, the largest island where tourism pressure is high and the ginger lily is abundant, did not change the population trends of either the native ground or canopy fauna. Indeed, studies suggest that land use adjacent to native forest remnants has a greater influence on the spread of invasive plants than outdoor activities, such as hiking^38,39^. Nonetheless, the number of SIE and FDE species decreased only on this island and *A. hamiltoni* declined more severely than any other species leaving it unclear whether other ground-dwelling species, for which detailed data are lacking, might be facing a similarly grave situation. This discrepancy could be due to the presence of already degraded and small habitat patches that cannot sustain viable populations of specialised species like FDEs and SIEs. Thus, some arthropod species may have already gone extinct due to historical events^2,26^ rather than current pressures. Indeed, recent work on ant assemblages in the Fijian archipelago suggested that the decline of endemic species started with the first human arrivals, there nearly 3000 years ago^22^.

At the same time, the marked differences among our three sampling periods caution against interpreting the observed patterns as a simple linear response to steadily increasing anthropogenic pressure. Broad syntheses have shown that insect trends are often highly heterogeneous across taxa, habitats and regions, with declines, increases and stable trajectories occurring simultaneously^12,31^ and they may be driven by a variety of factors^33^, including weather^40^, emphasizing that substantial temporal variability is itself a core feature of insect population dynamics.

Nevertheless, intensive conservation measures implemented over the past three decades in the Azores appear to have mitigated significant direct threats to native habitats and our results allow for cautious optimism regarding the resilience of endemic arthropods to indirect anthropogenic effects. Native forest patches on Terceira Island, which are among the largest in the Azores, seem to exhibit resilience against invasive species, further supporting the idea that habitat protection is crucial for the survival of endemic species. On the other hand, secondary forests of exotic tree species may serve as refuges for some forest specialist endemics, allowing for population stability^41,42^. However, there is no evidence of spatial rescue effects for isolated populations of rare endemic species^43^ and no protection fully excludes long-term disturbances from invasive plant encroachment and occasional human or livestock disturbances, nor does it mitigate the ongoing effects of climate change. Indeed, some endemics continue to show declines, with three FDEs—*Eupteryx azorica* Ribaut, 1941, *Cixius azoricus azoricus* Lindberg, 1954 leafhoppers, and the *Atlantocis gillerforsi* Israelson, 1985 beetle—not found in recent samples, and others, such as the spider *M. floresensis*, becoming increasingly rare.

Yet, while Triantis et al.^20^ predicted that 55-99% of FDEs would eventually face extinction, we found strong statistical evidence of decline in two species, with indications of decline in 25 additional species out of a total of 55 species (SIEs and FDEs combined). Of the 34 FDE species analysed by Triantis et al., 14 (~41%) showed any signs of decline—far below the predicted range, although across a narrow observational window. Moreover, 11 FDE species exhibited population increases, challenging the prevailing narrative of impending biodiversity loss. However, the observation that some SIE from Santa Maria Island are decreasing in abundance (see Supplementary Table 2.) highlights the fact that small native forest fragments may not be adequate to support the long-term persistence of some of the Azorean endemic species.

The limited information on the original arthropod fauna prevents us from ruling out the possibility that the lack of widespread decline among SIE and FDE species indicates that the Azorean fauna had already been impoverished by the time biodiversity research began in this archipelago, with many rare habitat specialists potentially extirpated^2^ or only persisting within relictual sites^42^. Nonetheless, based on our current knowledge, protecting native habitats appears key to the long-term survival of prospering species, while declining species may benefit from additional focused protection programs^44^.

As introduced species often drive endemic extinctions^45^, simultaneous increases in exotics and declines in endemics can be anticipated. However, despite reports of a clear increase in non-native species in other studies on Azorean arthropods^16,35^, we found little evidence that exotics are rapidly outcompeting natives or endemics. On the contrary, declines of comparable magnitudes were noted in the abundances of both endemics and exotics, with slightly (but not significantly) higher increases in exotics. While it is particularly reassuring that neither the abundance nor the species richness proportion of exotics changed in communities over this more than 20-year timespan, their biomass and biomass-based proportions did increase between the first and last sampling campaigns. While this solely impacted canopy-dwelling communities, the gains exotics exhibited indicate unfavourable changes for indigenous species. However, looking solely at richness or abundance, species replacements and patterns in assemblage turnover may remain hidden. Nevertheless, exotics decreased more in site occupancy than SIE and FDE species, suggesting a continuous influx of exotics with low colonization success from disturbed edges of the forest patches^46^. Although these pioneer individuals have the potential to get established and trigger processes that rewire island ecosystems^47^, in previous studies, neither Whittaker et al.^48^ nor Pozsgai et al.^47^ found clear evidence for functionally matching exotics outcompeting indigenous species in the Azores. Furthermore, within healthy forest remnants, like those on Terceira Island, the increase of exotics appears limited, while endemic arthropod populations have persisted well. It thus seems possible that long-term environmental shifts, including climate change, provide a greater threat than invasive non-native species.

### Limitations and future perspectives

While the discontinuous time series presents a limitation, the extensive temporal and spatial scale, combined with substantial sampling efforts, make the BALA data a uniquely well-specified basis for uncovering changing diversity patterns. The restricted temporal sampling window, which may reduce the ability to capture full seasonal variation in invertebrate communities across islands, can also be a weakness. Although the peak activity in the Azorean summer during which the sampling was conducted has balanced weather and allows relatively low phenological differences in communities, some temporal turnover cannot be excluded and may influence observed patterns of community composition. Moreover, the fact that we only could compare three distinct sampling periods renders the knowingly variable abundance^33^ trends less reliable. Yet, our sensitivity analysis, which showed minimal divergences in results with up to 20% random sample removal (see Methods and Supplementary Figures 11-13), particularly alongside with the little-changing species occurrences, confirms the robustness of the patterns we found. However, the relatively low number of species whose temporal patterns could be statistically tested underscores the urgency of establishing a large-scale and continuous monitoring system to assess long-term impacts on arthropod communities.

Several key questions remain unanswered. For instance, we know little about nuanced community changes, such as species replacement, or how species traits influence population trends. Future research should delve into the details driving these trends, considering species’ traits, environmental constraints, and biotic interactions. Additionally, understanding whether secondary or semi-natural habitats can mitigate biodiversity losses will be essential.

## Concluding remarks

The temporal patterns in our study did not consistently match the expectations of demise of insular arthropods, suggesting that predicted declines may not be uniformly expressed across all island contexts, particularly in conserved native forest habitats. They foster moderate optimism for the future of island biotas where habitats remain intact and highlight the importance of long-term monitoring and effective conservation strategies and the need for habitat protection to preserve obscure biodiversity.

However, one unfortunate conclusion from our work is that declines may still occur within protected areas, indicating that the quality or size of these preserves may not be sufficient to buffer all negative anthropogenic impacts and prevent biodiversity losses. Thus, our study has some significant implications. First, although habitat protection appears to be effective in at least the short run, some species require additional support from specific protection programs, such as recently implemented LIFE Beetles^44,49^ and LIFE Snails^50^ projects. Second, the presence of introduced arthropods does not appear to generally adversely affect indigenous species within Azorean native forest remnants; rather, the structural condition of the habitat is the key factor determining the persistence of indigenous arthropod communities. Therefore, plant invasions may pose a more serious threat than competition from other arthropods. Last but not least, if the natural capital of oceanic islands and the dependent ecosystem services are to be protected, monitoring both indigenous and introduced species, and regularly re-assessing community changes should be a priority^51^. Even simplified, regular monitoring systems utilising advanced technologies and focusing on selected indicator species would be adequate for establishing early warning systems to detect the establishment of potentially competitive species or significant declines in species of high conservation importance. This proactive approach, combined with targeted removal of invasive habitat-structuring plants and competing arthropod exotics, will be key to the success of conservation efforts in the Azores and other insular ecosystems.

## Methods

### Study sites and sampling methods

We used arthropod data collected from the Azorean archipelago (Fig. 1), using standardised sampling protocols under the umbrella of the ‘Biodiversity of Arthropods the Laurisilva of Azores’ (BALA) project^25,28,29^. In the course of the project, 30 sites of native Azorean humid forests^52^ were chosen for standardised arthropod sampling, repeated in three sampling campaigns: B1 (1999-2002), B2 (2010-2011), and B3 (2021-2023). The sites were spread across 15 native forest remnants, on seven out of the nine Azorean islands (from west to east: Flores, Faial, Pico, São Jorge, Terceira, São Miguel, and Santa Maria), those with remaining native vegetation. Summary data on the transects and remnants are available in Pozsgai et al.^25^. Major differences in Azorean arthropod communities are only between the cold (winter-spring) and hot seasons (summer autumn)^53^, and all of our samples were collected in the hot season, between July and September, when arthropod activity is at its peak.

To encompass a broad range of micro-habitats, we employed two primary sampling techniques. The first involved pitfall trapping, to effectively capture ground-dwelling (epigeal) arthropods. At each site, across a 150 m transect, 30 pitfall traps were evenly distributed, maintaining a separation of 5 m between each. Alternating preservative/attractive solutions of ethylene-glycol and Turquin’s solution (10 g chloral hydrate, 5 ml formalin, 5 ml acetic acid, added to 1 L of dark beer)^25,54^ were employed in these traps, maximising catch. Pitfall traps were active for two weeks in each sampling campaign.

The second technique, canopy beating, was employed to target canopy-dwelling (arboreal) arthropods. At each selected site, the beating was performed on the three, locally most abundant tree species, primarily focusing on native *Juniperus brevifolia* (Cupressaceae), *Erica azorica* (Ericaceae), *Ilex azorica* (Aquifoliaceae), *Laurus azorica* (Lauraceae), and *Vaccinium cylindraceum* (Ericaceae). For each of the three species, ten individual trees were randomly selected along the transect and the branches were beaten five times at the height of 1.5-2 m (Azorean humid forests have a median canopy height of ca. 13 m, rarely reaching 25 m^55^). All visually observable arthropods, except for mites (Acari) and springtails (Collembola), were sampled. Specimens were sorted into morphospecies and, where possible, identified to the biological species. Due to difficulties in their identification, Diptera and Hymenoptera (with the exception of Formicidae, which were included) were excluded from the dataset. Biogeographic origin, categorised as endemic to the Azores, native but not endemic to the Azores (termed as native for brevity), introduced (non-native, also termed as exotic), and of unknown origin, was assigned to each species (Ref.^25,27^ and references therein). Single-island endemics (SIE) were identified based on the dataset described in Ref.^25^ and the species list in the Supplementary Material of Ref.^20^ was used as the basis to identify strict forest-dependent endemics (FDE).

We also estimated the species-specific body mass for each non-lepidopteran species in our database. The body length of 3**−**10 individuals of each (morpho)species in an adult stage was measured and species-specific body mass was calculated based on published body length – body mass equations of the higher taxa (data available from Ref.^56^). Since adult and larval biomasses of Lepidoptera differ significantly, we excluded this group from the biomass-based analysis. Lepidoptera, however, were included in species counts and abundance data. To calculate sample-based biomass, we multiplied the number of collected individuals of each species occurring in the samples by their corresponding species-specific body mass value.

### Data analysis

We took two different approaches to explore potential arthropod declines in Azorean native forests.

First, we compared the changes at a community level and investigated whether there was a change between the first (B1) and second (B2), second and third/last (B3), and the first and third sampling events in any of three calculated measures: abundances, biomasses, and species richness. We used Spearman’s correlation test to estimate the interdependence of biomass and abundance values.

Whilst alternative methods, such as mixed effect models, are commonly used for comparing temporal data, given the complexity of the dataset and the limited temporal replication, we considered the calculation of effect sizes to be superior. Individual samples (i.e. one pitfall trap or the material from beating one tree) were pooled within each sampling transect, for each year when sampling was conducted, and the three measures were calculated for each of these pooled samples. Since the length between two sampling campaigns at the same place varied slightly, we did not directly compare the summarised measures from each sampling round but calculated a standardised yearly change in all three measures. To do so, at each site and for each measure, we subtracted the value of the later sampling from the earlier one and divided it by the number of years that passed between the two sampling events. This gave us the absolute yearly change in species richness, abundance, and biomass for each transect. Although this absolute value was used for further analysis, we also calculated the change as a percentage by dividing the absolute yearly change by the corresponding value (i.e. abundance, biomass, or species richness) of the previous sampling event. To estimate the magnitude of changes between sampling events and to test whether they were significant, we calculated Cohen D-s, using the cohens_d() function from the *rstatix* R package^57^. In-text categorisation of effect sizes (i.e. “negligible”, “small”, “moderate”, and “large”) followed that of Cohen^58^. We estimated the confidence intervals (CI) through a bootstrapping process with 10,000 iterations with the help of the *BootES* R package^59^ and compared the resulting values to a hypothetical zero change (a ‘zero change’ null model). We ran this analysis by including all data, and separately for subsets including species of each different biogeographic origin. Moreover, data for this analysis were separated by the sampling method. Thus, each of the three measures resulted in eight estimations of temporal patterns (pitfall trap – all species, pitfall trap – endemic species, pitfall trap – native species, pitfall trap – introduced species, beating – all species, beating – endemic species, beating – native species, beating – introduced species) between two sampling events.

As our second approach, we compared the changes at a species level, paying particular attention to the single-island endemics and strict forest-dependent endemics that may carry an extinction debt^20^. For this, we separated the species in our samples into two major groups: those were represented in at least five samples with four individuals and which were present in at least two sampling campaigns, and those that did not fulfil these criteria. For the first group, we used the same analysis as above to estimate whether abundance differences between two sampling events significantly differed from zero. We considered a species unsuitable for inclusion in the analysis (‘rare species’ henceforth) if they were represented with fewer than 30 individuals across all samples (i.e. fewer than the median number of individuals per species across all samples, when singletons and doubletons are removed), present in less than 1% of samples, or exhibiting scarcity or low presence (less than four individuals) in two entire sampling rounds. Whilst these values are inherently somewhat arbitrary, lower thresholds resulted in comparisons between extremely low abundances (1**−**2 individuals), or mean values calculated from unreasonably low sample sizes (three individuals) and lowered the power of our analysis. As these ‘rare species’ were neither abundant nor widespread enough to conduct a fully-fledged analysis, we only categorised the differences between sampling events as “no change”, “declines”, and “increases” based on how summary abundances differed between samplings. Since we wanted to avoid considering very little variability in abundances as changes, unless at least 20% change between two sampling events was observed, we considered the differences as “no change”. We conducted a sensitivity analysis to assess how altering this threshold influences our conclusions but, other than negligible numerical differences, they held until we reached 30%, and even after minor differences were present only in native species SIE/FDE species (Supplementary Figure 14). Although we did not conduct formal tests on the differences here, we compared whether the occurrence probability of categories (i.e., no change, declines and increases) differs significantly by undertaking a series of Fisher tests.

Fisher tests were also used to compare whether the site occupancy of species groups with a different biogeographic origin or conservation status (as defined by IUCN categorisation), has changed. For this, we recorded at how many sites each species occurred in each sampling event and compared them between sampling events. Changes were only considered when they reached a 20% difference. They then were categorised as “no change”, “declines”, and “increases” and Fisher tests were used to assess if site occupancy changes could be predicted from the biogeographic origin or SIE/FDE categorisation.

To assess the impact of island identity on the observed changes we removed one, two, and three islands in every combination from the original dataset and recalculated the changes in community measures (i.e., abundance, biomass, and richness) using the Cohen’s d calculation above.

For the sensitivity analysis, we randomly removed 1, 3, 5, 10, and 15 sites from the analysis and recalculated all community-level changes, using Cohen’s d, as above. We repeated each removal round 1000 times and compared the d values visually. All analyses were conducted in an R environment^60^, with the *dplyr*^61^, *ggplot2*^62^, and *reshape*^63^ packages used for data manipulation and plotting.

## Supplementary Information

Supplementary Information.

## Data Availability

The BALA dataset is available on the Global Biodiversity Facility’s (GBIF) website under the [https://doi.org/10.15468/rpdkx9] (https://doi.org/10.15468/rpdkx9) identifier. Basic information on the sampling sites and summary datasets used in the analysis are available on GP’s GitHub repository ([https://github.com/pozsgaig/BALA_diversity], (https://github.com/pozsgaig/BALA_diversity).

## Code availability

All code and data needed to reproduce the results presented in the article and supplementary material are available on the GitHub repository. An interactive version of the supplementary material is also downloadable from this repository.
