# Supplementary material for: Stability in the face of global decline: a 20-year study of arthropods in an oceanic archipelago
Source: Sci Rep. 2026 Jun 17;16:27703. doi: 10.1038/s41598-026-57146-5 (PMC13542152; doi:10.1038/s41598-026-57146-5)
Supplement: Supplementary file 1 [file 41598_2026_57146_MOESM1_ESM.html]

Stability in the Face of Global Decline: A 20-Year Study of Arthropods in an Oceanic Archipelago


# Stability in the Face of Global Decline: A 20-Year Study of Arthropods in an Oceanic Archipelago

Gabor Pozsgai^1\*^, Pedro Cardoso^2,3^, Simone Fattorini^4^, François Rigal^1,5^, Ana M.C. Santos^1,6,7^, Robert J. Whittaker^8,9^, Isabel R. Amorim^1,10^, Mário Boieiro^1,3,10^, Luís Borda-de-Água^11,12,13^, Ricardo Costa^1,3^, Luís Carlos Crespo^1,3^, Maria Teresa Ferreira^14^, Abrão Leite^15^, Sébastien Lhoumeau^1^, Thomas J. Matthews^1,16^, Jagoba Malumbres-Olarte^1,3^, Catarina Melo^1^, Guilherme Oyarzabal^1^, Fernando Pereira^1^, José A. Quartau^2^, Carla Rego^2,3,10^, Sérvio Ribeiro^17^, Alejandra Ros-Prieto^1^, Artur R.M. Serrano^2,3^, Kostas A. Triantis^18^, Rosalina Gabriel^1^ & Paulo A. V. Borges^1,10,19^

^1^University of the Azores, CE3C - Centre for Ecology, Evolution and Environmental Changes & CHANGE - Global Change and Sustainability Institute, Rua Capitão João d´Ávila, Pico da Urze, 9700-042, Angra do Heroísmo, Portugal.

^2^CE3C- Centre for Ecology, Evolution and Environmental Changes, CHANGE – Global Change and Sustainability Institute, Faculty of Sciences, University of Lisbon, Lisbon, Portugal.

^3^LIBRe – Laboratory for Integrative Biodiversity Research, Finnish Museum of Natural History, University of Helsinki, P.O.Box 17 (Pohjoinen Rautatiekatu 13), 00014 Helsinki, Finland.

^4^Department of Life, Health and Environmental Sciences, University of L’Aquila, Via Vetoio, 67100, L’Aquila, Italy.

^5^Institut Des Sciences Analytiques et de Physico Chimie pour L’environnement et les Materiaux UMR5254, Comité National de la Recherche Scientifique - University de Pau et des Pays de l’Adour - E2S UPPA, Pau, France.

^6^Terrestrial Ecology Group (TEG-UAM), Departamento de Ecología, Universidad Autónoma de Madrid, 28049 Madrid, Spain.

^7^Centro de Investigación en Biodiversidad y Cambio Global (CIBC-UAM), Universidad Autónoma de Madrid, 28049 Madrid, Spain.

^8^School of Geography and the Environment, University of Oxford, South Parks Road, Oxford OX1 3QY, UK.

^9^Center for Macroecology, Evolution and Climate, GLOBE Institute, University of Copenhagen, Universitetsparken 15, 2100 Copenhagen, Denmark.

^10^IUCN SSC Atlantic Islands Invertebrates Specialist Group, 9700-042 Angra do Heroísmo, Azores, Portugal.

^11^CIBIO, Centro de Investigação em Biodiversidade e Recursos Genéticos, InBIO Laboratório Associado, Campus de Vairão, Universidade do Porto, 4485-661 Vairão, Portugal

^12^CIBIO, Centro de Investigação em Biodiversidade e Recursos Genéticos, InBIO Laboratório Associado, Instituto Superior de Agronomia, Universidade de Lisboa, 1349-017 Lisboa, Portugal

^13^BIOPOLIS Program in Genomics, Biodiversity and Land Planning, CIBIO, Campus de Vairão, 4485-661 Vairão, Portugal

^14^Regional Secretariat of Environment and Climate Action, Project LIFE BEETLES (LIFE 18NAT/PT/000864), Rua do Galo n118, 9700-040, Angra do Heroísmo, Azores, Portugal.

^15^Rua Fernando Pessoa, nº99 R/C DTO 2765-483 Estoril, Portugal.

^16^GEES (School of Geography, Earth and Environmental Sciences) and Birmingham Institute of Forest Research, University of Birmingham, Birmingham, B15 2TT, UK.

^17^Laboratório de Ecologia do Adoecimento e Florestas, NUPEB, Instituto de Ciências Exatas e Biológicas, Universidade Federal de Ouro Preto, Campus Morro do Cruzeiro, 35400-000, Ouro Preto, MG, Brazil.

^18^Department of Ecology and Taxonomy, Faculty of Biology, National and Kapodistrian University of Athens, Athens GR-15784, Greece.

^19^IUCN SSC Species Monitoring Specialist Group, 9700-042 Angra do Heroísmo, Azores, Portugal.

# All biodiversity trends

Table S1. Changes of abundances, biomasses, and richnesses between sampling campaigns in Azorean native forests. Rows are colour coded according to the directionality of changes: blue hues represent significant declines, orange colours represent significant increases. Non-significant changes (e.g. when confidence intervals cross zero) are indicated with light grey rows. The magnitude of changes is indicated with colour depth, increasing from small
(representing a Cohan’s d value between 0.2 and 0.5), through medium (Cohan’s d value
between 0.5-0.7) to a large change (Cohan’s d > 0.7). Columns of the table can be sorted and filtered

Figure S1. Summary of changes in the abundance (A-B), biomass (C-D), and richness (E-F) proportions of endemic (END) and introduced (EXO) species between BALA sampling campaigns (B1-B3) in arboreal (A, C, E) and epigeal (B, D, F) communities. The proportion (from top to bottom) of endemic and exotic species are shown in the barplots. Dots in the thin lines using the same colour codes indicate the changes for each group. The arrows indicate the direction of comparison and their colour indicates the overall changes. Changes are standardised to time unit (year) and compared to a ‘zero change’ null model. Change was considered statistically not significant when confidence intervals crossed zero. The magnitude of change is indicated with colour depth, increasing from small (representing a Cohan’s d value between 0.2 and 0.5), through medium (Cohan’s d value between 0.5-0.7) to a large change (Cohan’s d > 0.7).

# Species trends

Table S2. Changes of abundances, biomasses, and richnesses between sampling campaigns in Azorean native forests. Rows are colour coded according to the directionality of changes: blue hues represent significant declines, orange colours represent significant increases. Non-significant changes (e.g. when confidence intervals cross zero) are indicated with light grey rows. The magnitude of changes is indicated with colour depth, increasing from small
(representing a Cohan’s d value between 0.2 and 0.5), through medium (Cohan’s d value
between 0.5-0.7) to a large change (Cohan’s d > 0.7). Columns of the table can be sorted and filtered

## List of species of high conseravtion importance

Table S3. Sum abundances and the number of occupies transects of Azorean arthropod species of high conservations interest, i.e. single island endemics (SIE) and forest dependent endemics (FDE), separated by sampling campaigns (BALA).

|  | | | | | | | Abundance | | | Occupancy | | |
| --- | --- | --- | --- | --- | --- | --- | --- | --- | --- | --- | --- | --- |
| MF | scientificname | class | order | family | FDE | SIE | BALA1 | BALA2 | BALA3 | BALA1 | BALA2 | BALA3 |
| MF107 | Atlantocis gillerforsi Israelson, 1985 | Insecta | Coleoptera | Ciidae | TRUE | FALSE | 1 | 4 | 0 | 1 | 3 | 0 |
| MF1088 | Drouetius borgesi sanctimichaelis Machado, 2009 | Insecta | Coleoptera | Curculionidae | FALSE | TRUE | 0 | 2 | 0 | 0 | 1 | 0 |
| MF1180 | Canariphantes relictus Crespo & Bosmans, 2014 | Arachnida | Araneae | Linyphiidae | FALSE | TRUE | 1 | 3 | 4 | 1 | 1 | 2 |
| MF1183 | Canariphantes junipericola Crespo & Bosmans, 2014 | Arachnida | Araneae | Linyphiidae | FALSE | TRUE | 1 | 0 | 5 | 1 | 0 | 2 |
| MF1188 | Tarphius wollastoni Crotch, 1867 | Insecta | Coleoptera | Zopheridae | TRUE | TRUE | 0 | 1 | 7 | 0 | 1 | 3 |
| MF1193 | Savigniorrhipis topographicus Crespo, 2013 | Arachnida | Araneae | Linyphiidae | FALSE | TRUE | 18 | 3 | 4 | 1 | 1 | 2 |
| MF12 | Cyclophora puppillaria granti (Prout, 1935) | Insecta | Lepidoptera | Geometridae | FALSE | TRUE | 41 | 13 | 2 | 2 | 2 | 2 |
| MF1211 | Calathus carvalhoi Serrano & Borges, 1986 | Insecta | Coleoptera | Carabidae | FALSE | TRUE | 0 | 0 | 8 | 0 | 0 | 1 |
| MF1217 | Tarphius furtadoi Borges & Serrano, 2017 | Insecta | Coleoptera | Zopheridae | TRUE | FALSE | 29 | 191 | 284 | 3 | 7 | 10 |
| MF128 | Pseudocaulotrupis parvus (Israelson, 1985) | Insecta | Coleoptera | Curculionidae | FALSE | TRUE | 3 | 4 | 2 | 2 | 2 | 1 |
| MF134 | Gibbaranea occidentalis Wunderlich, 1989 | Arachnida | Araneae | Araneidae | TRUE | FALSE | 1056 | 883 | 1217 | 23 | 26 | 28 |
| MF136 | Athous azoricus Platia & Gudenzi, 2002 | Insecta | Coleoptera | Elateridae | TRUE | FALSE | 0 | 0 | 26 | 0 | 0 | 1 |
| MF137 | Pinalitus oromii J. Ribes, 1992 | Insecta | Hemiptera | Miridae | TRUE | FALSE | 682 | 295 | 507 | 28 | 27 | 28 |
| MF138 | Athous pomboi Platia & Borges, 2002 | Insecta | Coleoptera | Elateridae | FALSE | TRUE | 1 | 13 | 1 | 1 | 2 | 1 |
| MF179 | Leucognatha acoreensis Wunderlich, 1992 | Arachnida | Araneae | Tetragnathidae | TRUE | FALSE | 479 | 514 | 715 | 26 | 27 | 28 |
| MF181 | Savigniorrhipis acoreensis Wunderlich, 1992 | Arachnida | Araneae | Linyphiidae | TRUE | FALSE | 3118 | 3692 | 4696 | 27 | 28 | 28 |
| MF208 | Lasaeola oceanica Simon, 1883 | Arachnida | Araneae | Theridiidae | TRUE | FALSE | 61 | 39 | 83 | 16 | 9 | 16 |
| MF222 | Trechus terrabravensis Borges, Serrano & Amorim, 2004 | Insecta | Coleoptera | Carabidae | TRUE | TRUE | 236 | 33 | 43 | 3 | 3 | 3 |
| MF244 | Alestrus dolosus (Crotch, 1867) | Insecta | Coleoptera | Elateridae | TRUE | FALSE | 143 | 160 | 70 | 8 | 11 | 6 |
| MF251 | Tarphius tornvalli Gillerfors, 1985 | Insecta | Coleoptera | Zopheridae | TRUE | TRUE | 377 | 115 | 32 | 4 | 4 | 4 |
| MF253 | Phloeostiba azorica (Fauvel, 1900) | Insecta | Coleoptera | Staphylinidae | TRUE | FALSE | 3 | 0 | 64 | 2 | 0 | 7 |
| MF255 | Cixius insularis Lindberg, 1954 | Insecta | Hemiptera | Cixiidae | FALSE | TRUE | 641 | 498 | 1404 | 4 | 4 | 4 |
| MF263 | Calathus lundbladi Colas, 1938 | Insecta | Coleoptera | Carabidae | FALSE | TRUE | 4 | 0 | 0 | 2 | 0 | 0 |
| MF288 | Hipparchia miguelensis (Le Cerf, 1935) | Insecta | Lepidoptera | Nymphalidae | FALSE | TRUE | 1 | 0 | 0 | 1 | 0 | 0 |
| MF295 | Cixius azopifajo azopifajo Remane & Asche, 1979 | Insecta | Hemiptera | Cixiidae | FALSE | TRUE | 204 | 694 | 616 | 4 | 4 | 4 |
| MF302 | Cedrorum azoricus caveirensis Borges & Serrano, 1993 | Insecta | Coleoptera | Carabidae | FALSE | TRUE | 60 | 8 | 0 | 2 | 1 | 0 |
| MF307 | Tarphius floresensis Borges & Serrano, 2017 | Insecta | Coleoptera | Zopheridae | TRUE | TRUE | 130 | 35 | 96 | 4 | 3 | 4 |
| MF312 | Acorigone acoreensis (Wunderlich, 1992) | Arachnida | Araneae | Linyphiidae | TRUE | FALSE | 73 | 88 | 321 | 16 | 18 | 20 |
| MF313 | Agyneta depigmentata Wunderlich, 2008 | Arachnida | Araneae | Linyphiidae | FALSE | TRUE | 73 | 24 | 14 | 2 | 4 | 2 |
| MF346 | Cedrorum azoricus azoricus Borges & A.Serrano, 1993 | Insecta | Coleoptera | Carabidae | TRUE | FALSE | 30 | 31 | 36 | 3 | 2 | 1 |
| MF4 | Porrhomma borgesi Wunderlich, 2008 | Arachnida | Araneae | Linyphiidae | TRUE | FALSE | 5 | 5 | 8 | 2 | 4 | 4 |
| MF416 | Cixius azoricus azoricus Lindberg, 1954 | Insecta | Hemiptera | Cixiidae | TRUE | FALSE | 3 | 0 | 0 | 2 | 0 | 0 |
| MF421 | Walckenaeria grandis (Wunderlich, 1992) | Arachnida | Araneae | Linyphiidae | TRUE | FALSE | 14 | 5 | 2 | 4 | 5 | 2 |
| MF431 | Cheiracanthium floresense Wunderlich, 2008 | Arachnida | Araneae | Cheiracanthiidae | FALSE | TRUE | 28 | 32 | 32 | 3 | 3 | 3 |
| MF439 | Notothecta dryochares (Israelson, 1985) | Insecta | Coleoptera | Staphylinidae | TRUE | FALSE | 24 | 39 | 79 | 9 | 12 | 19 |
| MF442 | Minicia floresensis Wunderlich, 1992 | Arachnida | Araneae | Linyphiidae | TRUE | FALSE | 80 | 12 | 29 | 9 | 6 | 9 |
| MF46 | Drouetius borgesi borgesi (Machado, 2009) | Insecta | Coleoptera | Curculionidae | FALSE | TRUE | 17 | 6 | 4 | 3 | 1 | 4 |
| MF465 | Eupteryx azorica Ribaut, 1941 | Insecta | Hemiptera | Cicadellidae | TRUE | FALSE | 1 | 1 | 0 | 1 | 1 | 0 |
| MF490 | Tarphius sp. | Insecta | Coleoptera | Zopheridae | TRUE | FALSE | 2 | 27 | 0 | 1 | 2 | 0 |
| MF5 | Rugathodes acoreensis Wunderlich, 1992 | Arachnida | Araneae | Theridiidae | TRUE | FALSE | 982 | 1432 | 1717 | 26 | 29 | 29 |
| MF526 | Neon acoreensis Wunderlich, 2008 | Arachnida | Araneae | Salticidae | TRUE | FALSE | 4 | 10 | 6 | 3 | 6 | 5 |
| MF557 | Strophingia harteni Hodkinson, 1981 | Insecta | Hemiptera | Liviidae | TRUE | FALSE | 258 | 209 | 135 | 10 | 9 | 10 |
| MF568 | Phloeosinus gillerforsi Bright, 1987 | Insecta | Coleoptera | Curculionidae | TRUE | FALSE | 3 | 1 | 1 | 2 | 1 | 1 |
| MF587 | Cixius azopifajo azojo Remane & Asche, 1979 | Insecta | Hemiptera | Cixiidae | FALSE | TRUE | 453 | 98 | 89 | 4 | 4 | 4 |
| MF589 | Acorigone zebraneus Wunderlich, 2008 | Arachnida | Araneae | Linyphiidae | FALSE | TRUE | 7 | 0 | 0 | 2 | 0 | 0 |
| MF613 | Cixius azopifajo azofa Remane & Asche, 1979 | Insecta | Hemiptera | Cixiidae | FALSE | TRUE | 220 | 61 | 34 | 3 | 2 | 4 |
| MF64 | Tarphius serranoi Borges, 1991 | Insecta | Coleoptera | Zopheridae | TRUE | TRUE | 53 | 63 | 5 | 2 | 2 | 1 |
| MF7 | Cixius azoterceirae Remane & Asche, 1979 | Insecta | Hemiptera | Cixiidae | TRUE | TRUE | 830 | 655 | 729 | 8 | 8 | 8 |
| MF70 | Tarphius pomboi Borges, 1991 | Insecta | Coleoptera | Zopheridae | TRUE | TRUE | 47 | 132 | 22 | 2 | 2 | 2 |
| MF76 | Tarphius rufonodulosus Israelson, 1984 | Insecta | Coleoptera | Zopheridae | TRUE | TRUE | 2 | 5 | 6 | 1 | 2 | 2 |
| MF77 | Pseudechinosoma nodosum Hustache, 1936 | Insecta | Coleoptera | Curculionidae | TRUE | FALSE | 11 | 13 | 6 | 6 | 6 | 4 |
| MF8 | Aphrodes hamiltoni Quartau & Borges, 2003 | Insecta | Hemiptera | Cicadellidae | TRUE | FALSE | 225 | 88 | 84 | 24 | 24 | 24 |
| MF85 | Tarphius depressus Gillerfors, 1985 | Insecta | Coleoptera | Zopheridae | TRUE | TRUE | 2 | 0 | 3 | 1 | 0 | 2 |
| MF925 | Cixius azomariae Remane & Asche, 1979 | Insecta | Hemiptera | Cixiidae | FALSE | TRUE | 52 | 40 | 32 | 2 | 2 | 2 |
| MF928 | Cheiracanthium jorgeense Wunderlich, 2008 | Arachnida | Araneae | Cheiracanthiidae | FALSE | TRUE | 11 | 7 | 1 | 4 | 2 | 1 |

## Rare species with not enough data for analysis

84 were abundant enough to be included in the full analysis.

Altogether, 186 (58.31%), species occurred in less transects in B3 than in B1, 37 (11.6%) showed no change and 96 (30.09%) occured in more transects.

Table S4. Number of species showing different abundance trends across diferent biogeographic origin.

|  | END | NAT | EXO | UnK |
| --- | --- | --- | --- | --- |
| Decrease | 36 | 31 | 71 | 48 |
| No change | 5 | 14 | 14 | 4 |
| Increase | 20 | 21 | 43 | 12 |

Table S5. Proportion of species showing different abundance trends across diferent biogeographic origin.

|  | END | NAT | EXO | UnK |
| --- | --- | --- | --- | --- |
| Decrease | 59.016393 | 46.96970 | 55.46875 | 75.00 |
| No change | 8.196721 | 21.21212 | 10.93750 | 6.25 |
| Increase | 32.786885 | 31.81818 | 33.59375 | 18.75 |

There is no evidence that the abundance of endemic species increases in greater proportion than that of introduced (Fisher’s Test with simulated p-values; p = 0.529, odds ratio = 1.045, 95% CI: 0.523 - Inf).There is no evidence that the abundance of endemic species declines in greater proportion than that of introduced (Fisher’s Test with simulated p-values; p = 0.938, odds ratio = 1.619, 95% CI: 0 - 3.111).

Table S6. Number of species showing different abundance trends separated whether they are of high conservation importnace.

|  | FALSE | TRUE |
| --- | --- | --- |
| Decrease | 161 | 25 |
| No change | 33 | 4 |
| Increase | 84 | 12 |

Table S7. Number of species showing different abundance trends separated whether they are forest-dependent endemics.

|  | FALSE | TRUE |
| --- | --- | --- |
| Decrease | 174 | 12 |
| No change | 36 | 1 |
| Increase | 89 | 7 |

# Changes in the number of sites occupied

We recorded the presence of each species at each site during each sampling campaign and examined whether the number of sites occupied by each species increased or decreased between campaigns (approximately every 10 years).

Altogether (including all species) 205 species occurred in less transects in B3 than in B1, 65 showed no change and 133 occured in more transects.

Table S8. Changes in site occupancy of species with diferent biogeographic origin, including all species.

|  | END | NAT | EXO | UnK |
| --- | --- | --- | --- | --- |
| Decrease | 34 | 41 | 80 | 50 |
| No change | 25 | 15 | 19 | 6 |
| Increase | 32 | 33 | 54 | 14 |

Of those spcies which were excluded from the full statistical analysis, 171 (0.54%), species occurred in less transects in B3 than in B1, 53 (0.17%) showed no change and 95 (0.3%) occured in more transects.

# The influence of islands on biodiversity trends

## The influence of island removal on abundances

Figure S2. Results of the simulated island removal on the overall abundance changes between B1 and B3. Cohen d-s and corresponding confidence intervals are shown in black markings. Dashed vertical red line indicates the Cohen d calculated before any island removal and pink shaded area indicate confidence intervals

Figure S3. Results of the simulated island removal on the abundance changes of endemic species between B1 and B3. Cohen d-s and corresponding confidence intervals are shown in black markings. Dashed vertical red line indicates the Cohen d calculated before any island removal and pink shaded area indicate confidence intervals

Figure S4. Results of the simulated island removal on the abundance changes of introduces species between B1 and B3. Cohen d-s and corresponding confidence intervals are shown in black markings. Dashed vertical red line indicates the Cohen d calculated before any island removal and pink shaded area indicate confidence intervals

## The influence of island removal on biomass

Figure S5. Results of the simulated island removal on the overall biomass changes between B1 and B3. Cohen d-s and corresponding confidence intervals are shown in black markings. Dashed vertical red line indicates the Cohen d calculated before any island removal and pink shaded area indicate confidence intervals

Figure S6. Results of the simulated island removal on the biomass changes of endemic species between B1 and B3. Cohen d-s and corresponding confidence intervals are shown in black markings. Dashed vertical red line indicates the Cohen d calculated before any island removal and pink shaded area indicate confidence intervals

Figure S7. Results of the simulated island removal on the biomass changes of introduces species between B1 and B3. Cohen d-s and corresponding confidence intervals are shown in black markings. Dashed vertical red line indicates the Cohen d calculated before any island removal and pink shaded area indicate confidence intervals

## The influence of island removal on species richness

Figure S8. Results of the simulated island removal on the overall species richness changes between B1 and B3. Cohen d-s and corresponding confidence intervals are shown in black markings. Dashed vertical red line indicates the Cohen d calculated before any island removal and pink shaded area indicate confidence intervals

Figure S9. Results of the simulated island removal on the species richness changes of endemic species between B1 and B3. Cohen d-s and corresponding confidence intervals are shown in black markings. Dashed vertical red line indicates the Cohen d calculated before any island removal and pink shaded area indicate confidence intervals

Figure S10. Results of the simulated island removal on the species richness changes of introduced species between B1 and B3. Cohen d-s and corresponding confidence intervals are shown in black markings. Dashed vertical red line indicates the Cohen d calculated before any island removal and pink shaded area indicate confidence intervals

# Sample removal sensitivity analysis

Figure S11. The effect of simulated sample removal on the measured effect sizes between B1 and B3 sampling campaigns. Effects are separated by datasets and sampling methods, and differently coloured lines indicate the effects on all (ALL), endemic (END), introduces (EXO), and native (NAT) species.

Figure S12. The effect of simulated sample removal on the measured effect sizes between B1 and B2 sampling campaigns. Effects are separated by datasets and sampling methods, and differently coloured lines indicate the effects on all (ALL), endemic (END), introduces (EXO), and native (NAT) species.

Figure S13. The effect of simulated sample removal on the measured effect sizes between B2 and B3 sampling campaigns. Effects are separated by datasets and sampling methods, and differently coloured lines indicate the effects on all (ALL), endemic (END), introduces (EXO), and native (NAT) species.

# Sensitivity analysis for decline threshold value

In this study, we used a 20% difference in abundance between the first and last sampling campaigns as a threshold to distinguish between change in abundance or no change. Here, we show how choosing different values would influence the number of species considered as declining, experiencing no change, or increasing.

Figure S14. The dependence of how species trends are categorised on the definition of the threshold value for all (ALL), endemic (END), native (NAT), introduced species (EXO) and thos of high conservation importance (CONS), i.e. single island endemics and forest-dependent endemics together.
